# Bioactivity of *Juglans regia* kernel extracts optimized using response surface method and artificial neural Network-Genetic algorithm integration

**DOI:** 10.1038/s41598-025-93499-z

**Published:** 2025-03-15

**Authors:** Ayşenur Gürgen

**Affiliations:** Faculty of Engineering and Natural Sciences, Department of Industrial Engineering, Osmaniye Korkut Ata University, Osmaniye, 80000 Turkey

**Keywords:** Bioactivity, Extract optimization, *Juglans regia*, Walnut, Biochemistry, Biotechnology, Developmental biology, Chemistry, Engineering

## Abstract

In this study, the biological activities of the extracts obtained under optimum extraction conditions of the kernel part of *Juglans regia* L. were determined. Two different methods, Response Surface Method (RSM) and Artificial Neural Network-Genetic Algorithm (ANN-GA) integration, were used for optimization. The antioxidant capacity of the extracts obtained under the extract conditions suggested by the two methods was evaluated by Rel Assay kits, DPPH and FRAP methods. Anticholinesterase activities of the optimized extracts were measured by the action of acetylcholinesterase and butyrylcholinesterase enzymes. Antiproliferative effects of the extracts were tested on A549 lung cancer cell line. Phenolic compounds were analyzed by LC-MS/MS. It was determined that both extracts exhibited strong activities against A549 lung cancer cell line depending on the concentration increase. In addition, it was determined that both extracts exhibited acetyl and butyrylcholinesterase inhibition activity close to galantamine used as a standard. In both extracts, 13 compounds including gallic acid, catechinhyrate, 4-hydroxybenzoic acid, caffeic acid, vanillic acid, syringic acid, 2-hydoxycinamic acid, resveratrol, myricetin, quercetin, kaempferol, protocatechuic acid and 2-hyroxy1,4 naphthaquinone were identified. It was determined that the extract obtained under the conditions predicted by ANN-GA exhibited higher activities in general.

## Introduction

Medicinal plants have been used as a source of healing among the people for centuries. These plants play an important role in the treatment of various health problems thanks to the biologically active compounds they contain^1^. Plants have an important place in both traditional medicine and modern pharmaceutical research, showing properties such as antioxidant, antimicrobial and anti-inflammatory^2^. Today, scientific studies on medicinal plants reveal the health benefits of these plants in more detail and contribute to the development of new treatment methods^3^.

*Juglans regia* (walnut tree) is a large tree species that grows widely throughout the world, especially in the Mediterranean region, and is known for its fruit. The inner part of the walnut, the kernel, is a valuable source of nutrients for health. This part is characterized by its rich fat content and is particularly high in omega-3 fatty acids. In addition, walnut kernels are rich in protein, fiber, vitamins (especially vitamins E and B) and minerals. These nutrients support heart health, reduce inflammation and energize the body^4,5^. Walnuts have a strong antioxidant effect thanks to the polyphenols, flavonoids and other antioxidants they contain, which protect cells from the harmful effects of free radicals. In addition, the walnut inside is beneficial in reducing inflammation in the body and strengthening the immune system, as it contains compounds with anti-inflammatory properties^6,7^. Research has proven that walnut kernels can be beneficial in the treatment of neurological diseases, especially degenerative brain diseases such as Alzheimer’s. In addition, walnuts are of great importance in the field of health, as they have the potential to balance cholesterol levels, regulate blood sugar and fight chronic diseases such as cancer^8,9^. Walnuts are not only a nutritious food, but also an important herbal resource used in traditional and modern medicine^10^.

One of the most important steps in the utilization of plant resources is the extraction process. Optimizing extraction conditions is critical to maximize process efficiency by increasing the yield and purity of the desired compounds. in this context, response surface method (RSM) offers parametric analyses, while artificial intelligence methods such as artificial neural networks (ANNs) and artificial intelligence methods such as ANN-Genetic Algorithm (GA) integration combine the learning and modeling capabilities of ANNs with the global optimization capacity of GA to enable parameter optimization of complex systems with higher accuracy and efficiency. It has been reported that which method predicts with higher accuracy may vary according to the parameters of the study and problem dynamics^11^. The aim of this study is to maximize the utilization of the kernel part of *Juglans regia*. For this purpose, the study was designed in 4 steps.

(i) Preparation of extracts of kernel parts of walnut under 27 different conditions and determination of total antioxidant status (TAS) values of these extracts.
(ii) Optimization with both RSM and ANN-GA integration methods using the obtained data.
(iii) Obtaining the extracts under the optimum conditions prescribed by the methods.
(iv) Determination of the biological activities of the optimum extracts obtained.

## Material and method

Walnut samples used in the study were collected from Kahramanmaraş (Turkey). The kernel part of the walnut was removed from the hard part and dried in a Dalle SS-06 A fruit dryer at 40 ˚C for about 3 h. In the study, 10 g samples were used separately for all extracts. The dry samples were then pulverized in a mechanical grinder. Extraction procedures were performed from the powder samples.

### Chemicals

The solvent (Ethanol) used in this study was provided by Sigma-Aldrich (Saint-Quentin Fallavier, France). In addition, all pure chemicals used for phenolic analysis in the study were provided by Sigma-Aldrich (Steinheim, Germany). Trypsin-EDTA, phosphate buffer, chloride (FeCl_3_), dimethyl sulfoxide were provided by Sigma-Aldrich (MO, USA). TAS and TOS kits were provided by Megatıp, Gaziantep (Turkey). A549 (ccl-185) cell line was provided by ATTC (American Type Culture Collection).

### Method of extraction procedure

The extraction procedure was performed according to a full factorial experimental design. Three different parameters, namely extraction temperature, extraction time, and ethanol/water ratio, were selected, and extraction procedures were performed using three levels of these parameters. Twenty-seven different experiments were performed in the Soxhlet apparatus at 45, 55, and 65˚C extraction temperatures, 5, 10, and 15-hour extraction times, and 0, 50, and 100% ethanol/water conditions. The data obtained were optimized by both the RSM and ANN-GA integration, which is an artificial intelligence method.

### RSM

In this study, RSM was used for optimization. Extraction temperature, extraction time and ethanol/water ratio were selected as the independent variables of the study. The response variable was determined as the total antioxidant activity (TAS) value of the obtained extract.

The optimization process was carried out using Design Expert 13 software with a second-order polynomial response as:

$$\:{Y}_{k}={\beta\:}_{k0}+\sum\:_{i=1}^{n}{\beta\:}_{ki}{x}_{i}+\sum\:_{i=1}^{n}{\beta\:}_{kii}{x}_{i}^{2}+\sum\:_{i=1}^{n-1}\sum\:_{j=i+1}^{n}{\beta\:}_{kij}{x}_{i}{x}_{j}$$

where $\:{Y}_{k}$ was response variable ($\:{Y}_{i}$ was TAS value of extract); $\:{x}_{i}$ was coded process variables ($\:{x}_{1}$ was extraction temperature, $\:{x}_{2}$ was extraction time, and $\:{x}_{3}$ was ethanol/water ratio) and $\:{\beta\:}_{k0}$ is the value of fitted response at the design center point, respectively.

The suitability of the model was tested using coefficient of determination (R^2^), ANOVA analysis and *p-*values. Critical points were calculated from the derivatives of the model to optimize the response variable. In addition, three-dimensional surface plots were prepared to visualize the effects of independent variables. These plots were used to better understand the effects of variables on the response.

### ANN-GA

**Fig. 1:**
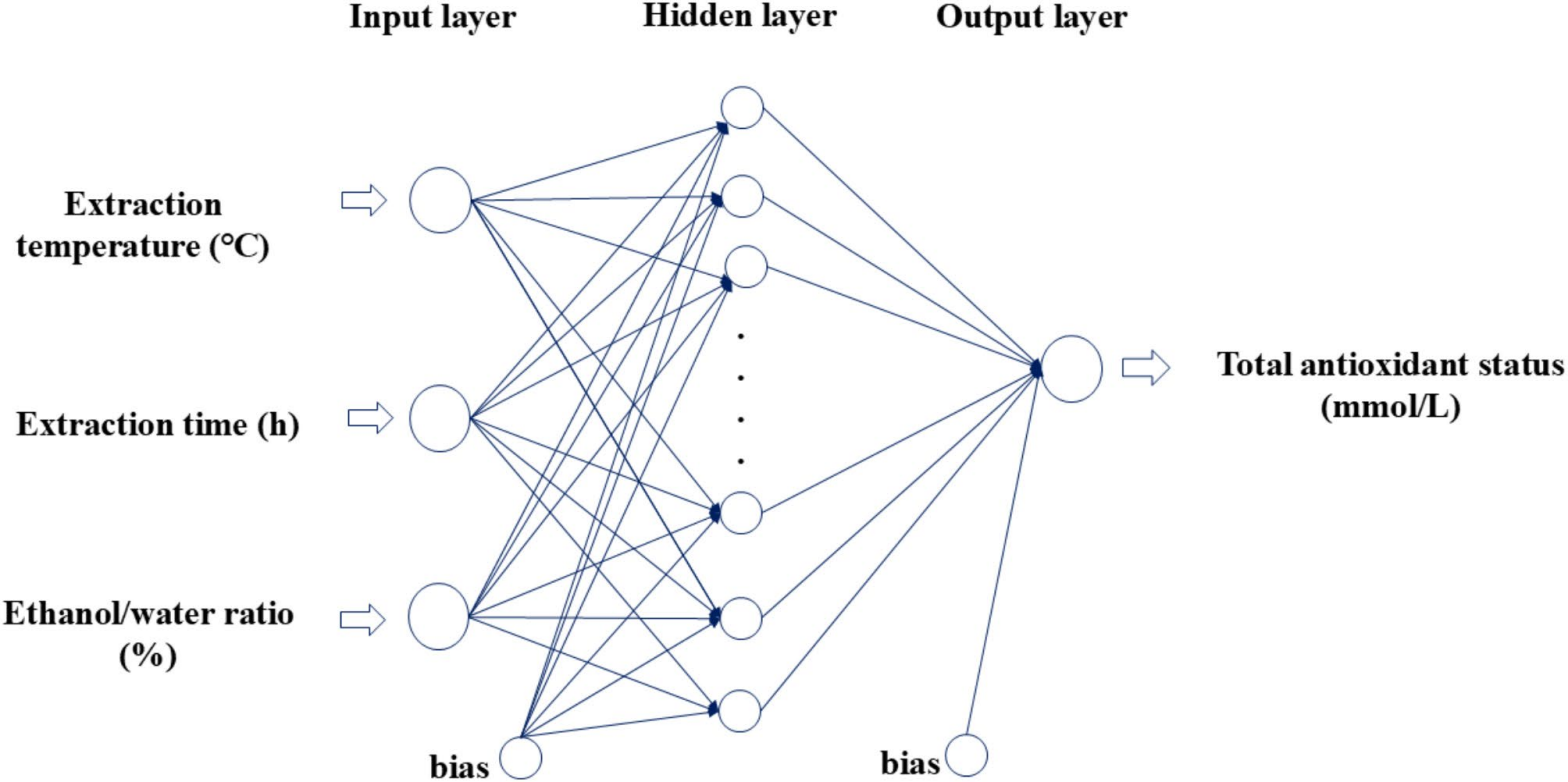
Layers of the ANN model.

Modeling was performed by the ANN method. The inputs of the model were extraction temperature, extraction time, and ethanol/water ratio, while the output was used as TAS value. The layers of the ANN used in the study are given in Fig. 1.

The data obtained from the experimental studies were used 80% for training, 10% for validation, and 10% for testing. The Levenberg-Marquardt (LM) algorithm was used for the learning process. 20 different (1:20) numbers of hidden neurons were compared to determine the optimal network. The learning coefficient and momentum coefficient were chosen as 0.5, the maximum number of iterations as 500, the number of validation checks as 50, and the error value as 1 × 10^− 5^. In this study, a total of 1000 training sessions were performed for each model.

In the study, the mean square error (MSE) and mean absolute percentage error (MAPE) were used as performance indicators of the developed models. MSE and MAPE were calculated according to following Eqs. (1) and (2);

1 $$\:\text{M}\text{S}\text{E}=\:\frac{1}{\text{n}}\sum\:_{\text{i}=1}^{\text{n}}{\left({\text{e}}_{\text{i}}-{\text{p}}_{\text{i}}\right)}^{2}\:$$

2 $$\:\text{M}\text{A}\text{P}\text{E}=\frac{1}{\text{n}}\sum\:\left|\frac{{\text{e}}_{\text{i}}-{\text{p}}_{\text{i}}}{{\text{e}}_{\text{i}}}\right|\text{*}100$$

where$\:,\:\text{e}$ is the experimental result, $\:\text{p}\:$is the prediction result, and $\:\text{n}$ is the number of samples.

The optimization procedure was performed using GA. Studies were carried out for different population numbers, and the roulette wheel technique was used for selection. For crossover, the single-point crossover method was used. The appropriate number of iterations was determined by analyzing the convergence graphs. Each optimization run was repeated at least 60 times to produce results very close to the global optimum.

### Extraction for bioactivity

The extracts showing the highest biological activity were obtained under the optimum conditions determined by two different methods. These conditions were determined as 53.612 ˚C temperature, 6.432 h duration, and 41.885 ethanol/water ratio according to the OE-RSM method and 57.123 ˚C temperature, 12.681 h duration, and 58.680 ethanol/water ratio according to OE-ANN-GA integration. These optimum conditions were applied with a computer-aided Gerhardt SOX-414 device, and biological activity was tested on the extracts obtained under these conditions.

### Antioxidant activity tests

#### Total antioxidant and oxidant analysis

The total antioxidant capacity of the optimized extracts of the kernel was measured using the Rel Assay TAS kit. The results obtained are expressed in mmol trolox equivalent/L. Total oxidant status was determined by Rel Assay TOS kit, and the results were reported as µmol hydrogen peroxide equivalent/L^12,13^. Oxidative stress index (OSI) was determined by the percentage calculation method by proportioning TOS values to TAS values^14^.

#### DPPH free radical scavenging activity

The study solution was prepared from the kernel crude extract by dissolving it in dimethyl sulfoxide (DMSO) to have a concentration of 1 mg/mL. A 1 mL aliquot of this solution was then combined with 160 µL of a 0.267 mM DPPH solution (prepared in 4 mL of 0.004% methanol), and the mixture was left to incubate for 30 min in the dark at room temperature. Following incubation, the absorbance was measured at 517 nm. The results were reported as milligrams of Trolox Equivalent per gram of extract^15^.

#### Ferric reducing antioxidant power assay

A 100 µL stock solution was prepared from optimized extracts of kernel and mixed with 2 mL of FRAP reagent. Then, 300 mM acetate buffer (pH 3.6), 40 mM HCl and 20 mM FeCl₃-6 H₂O solution were prepared with 10 mM 2,4,6-tris(2-pyridyl)-S-triazine solution and mixed with FRAP solution (10:1:1 ratio). This mixture was incubated at 37 °C for 4 min. After incubation, absorbance was measured at 593 nm. The results obtained were reported as mg Trolox Equivalent/g extract^15^.

#### Enzyme inhibition tests

The anticholinesterase activities of optimized extracts of kernel were evaluated based on the Ellman method^16^. Galantamine was used as a reference substance in this study. Stock solutions were prepared from the kernel extracts at different concentrations between 200-3.125 µg/mL. First, 130 µL of 0.1 M pH = 8 phosphate buffer was added to the microplate, followed by 10 µL of stock solution and 20 µL of AChE or BChE enzyme solutions, respectively, and incubated in the dark at 25°C for 10 min. After incubation, 20 µL DTNB solution (5,5”-dithiobis-(2-nitrobenzoic acid)) and 20 µL substrate (acetylcholine iodide or butyrylcholine iodide) were added and absorbance was measured at 412 nm. Enzyme inhibition rates and IC50 values were calculated and reported in µg/mL.

#### Antiproliferative activity

The antiproliferative effect of optimized extracts of kernel was evaluated on A549 lung cancer cell line. For this purpose, stock solutions were prepared at concentrations of 25, 50, 100 and 200 µg/mL. After 70–80% confluency of the cells, the cells were detached with 3.0 mL Trypsin-EDTA solution (Sigma-Aldrich, MO, USA) and seeded in plates. After seeding, cells were incubated for 24 h. Then, stock solutions were added to the cells and incubated for another 24 h. After incubation, the supernatants were removed from the growth medium and replaced with 1 mg/mL MTT solution and incubated at 37 °C until a purple precipitate formed. Finally, dimethyl sulfoxide (DMSO) (Sigma-Aldrich, MO, USA) was added to the MTT solution and absorbance was measured at 570 nm with an Epoch spectrophotometer (BioTek Instruments, Winooska, VT)^17^.

#### Phenolic analysis

Phenolic compounds of the optimized extracts of kernel were analyzed by LC-MS/MS. In this analysis, 24 standard compounds present in kernel extracts were screened. Analysis of phenolic compounds was carried out using Shimadzu LC-MS/MS-8030 instrument. Inertsil ODS 4 column (2 μm, 2.1 × 50 mm) was preferred for separation. The analysis was carried out in binary gradient mode and the flow rate was set as 0.4000 mL/min. Water containing 0.1% formic acid (Mobile Phase A) and methanol containing 0.1% formic acid (Mobile Phase B) were used as mobile phases. The B mobile phase concentration was initially determined as 5.0% and the B curve was set as 0. The maximum pressure limit was determined as 660 bar. SIL-20ACXR model autosampler was used for automatic injection of samples. The column temperature was fixed at 40 °C with CTO-10ASvp model column oven and the maximum temperature was set as 85 °C.

### Statistical analysis

‘SPSS 21.0 for Windows’ program was used in the statistical analysis of all of the analyses performed within the scope of this study. Since there were more than two groups in the tests studied, Simple Variance Analysis (BVA) was performed to determine the difference between the groups; Duncan test was applied at the confidence level (α = 0.05) to determine the difference between the groups. In the antioxidant activity section, t-test was applied since there were only two different groups.

## Results and discussions

### Optimization of extraction conditions

The aim of this study was to optimize the extraction conditions in order to make maximum use of walnut kernel. Therefore, the extraction conditions were optimized to maximize the total antioxidant potential of the extracts. TAS values obtained after the experimental study are given in Table 1.

**Table 1:** TAS values of the extracts obtained in the study.

Experiment number	Extraction temperature (°C)	Extraction time (h)	Ethanol/water ratio (%)	TAS (mmol/L)
1	45	5	0	8.895 ± 0.047^e^
2	45	10	0	9.377 ± 0.029^h^
3	45	15	0	7.717 ± 0.041^c^
4	45	5	50	8.873 ± 0.041^e^
5	45	10	50	9.419 ± 0.044^h^
6	45	15	50	7.694 ± 0.027^c^
7	45	5	100	8.876 ± 0.019^e^
8	45	10	100	9.382 ± 0.029^h^
9	45	15	100	7.695 ± 0.041^c^
10	55	5	0	10.353 ± 0.039^i^
11	55	10	0	12.387 ± 0.036^j^
12	55	15	0	9.087 ± 0.060^g^
13	55	5	50	10.375 ± 0.034^i^
14	55	10	50	12.393 ± 0.029^j^
15	55	15	50	9.008 ± 0.063^f^
16	55	5	100	10.352 ± 0.041^i^
17	55	10	100	12.401 ± 0.027^j^
18	55	15	100	9.023 ± 0.035^fg^
19	65	5	0	7.159 ± 0.032^b^
20	65	10	0	8.102 ± 0.057^d^
21	65	15	0	6.690 ± 0.036^a^
22	65	5	50	7.146 ± 0.043^b^
23	65	10	50	8.105 ± 0.051^d^
24	65	15	50	6.685 ± 0.052^a^
25	65	5	100	7.132 ± 0.035^b^
26	65	10	100	8.128 ± 0.034^d^
27	65	15	100	6.655 ± 0.045^a^

^a^Means having the different superscript letter(s) in the same column are significantly different (*p* < 0.05) according to Duncan’s multiple range test.

In this experimental study, the effect of extraction temperature, time, and ethanol/water ratio on the total antioxidant capacity (TAS) of walnut kernel was investigated, and the optimum conditions were determined. It was observed that the mean values between the studied groups were statistically significantly different (*p* < 0.05). The highest TAS values were obtained from extracts obtained from 55 °C temperature, 50% ethanol/water ratio, and 10 h of extraction conditions. It was observed that moderate temperatures and times were ideal for the dissolution of phenolic compounds, while higher temperatures (65 °C) and longer times (15 h) caused thermal degradation of these compounds. In terms of solvent ratio, a 50% ethanol/water mixture gave optimum results, while the use of pure water (0% ethanol) or pure ethanol (100%) resulted in lower TAS values. Overall, optimizing these parameters in a balanced manner plays a critical role in enhancing antioxidant capacity.

In the study, two different optimization methods were used using the data obtained from the experimental studies. In the optimization with RSM, linear, 2FI, quadratic and cubic regression models were established, and the quadratic model was selected due to the highest R² (coefficient of determination) value. R² shows the capacity of the model to explain the response of the independent variables. A high R² (e.g. ≥ 0.90) indicates that the model is suitable. In this study, the R² of the model was found to be 0.933.

The quadratic polynomial equation created as a result of the multiple regression analysis to determine the TAS values of J. regia’s kernel is shown below.

$$\:TAS=11.71-0.673\:{X}_{1}-0.006{\:X}_{2}-0.494\:{X}_{3}+0.001\:{{X}_{1}X}_{2}+0.177\:{X}_{1}{X}_{3}-0.006\:{X}_{2}{X}_{3}-2.61\:{X}_{1}^{2}+0.001{\:X}_{2}^{2}-1.67{\:X}_{3}^{2}\:\:\:\:\:\:\:\:\:\:\:$$

In the equation $\:{X}_{1}$, $\:{X}_{2}$ ve $\:{X}_{3}$ represent extraction temperature, extraction time and ethanol/water ratio, respectively.

**Fig. 2:**
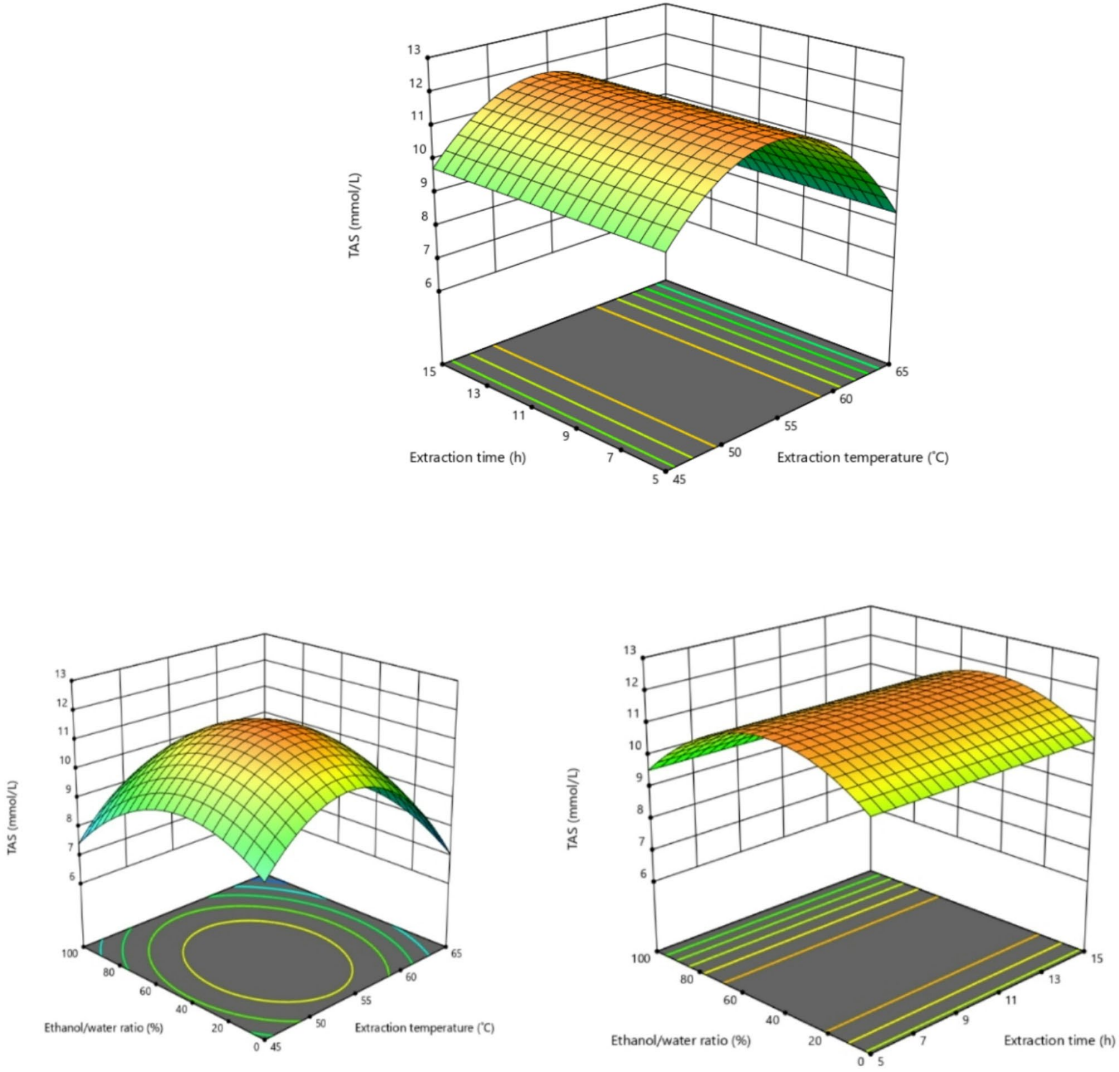
Response surface plots of TAS of *Juglans regia*’s kernel.

Response surface plots of TAS of *J. regia*’s kernel were shown at Fig. 2.

The TAS value of *J. regia*’s kernel extracts is mostly affected by the extraction temperature and ethanol/water ratio among the extraction conditions studied (statistically *p* < 0.05), and less affected by the extraction time. According to RSM optimization, the optimum conditions were predicted as 53.612 ˚C temperature, 6.432 h duration, and 41.885% ethanol/water ratio.

In the optimization phase with artificial intelligence methods, the data obtained from the experimental studies were modeled with ANN, and after the best model was selected, optimization was performed with GA. Among the models, the architecture of the best prediction model was found to be 3-4-1. In other words, the prediction model produced using 4 hidden neurons was selected as the best model. The performance of the best model is given in Table 2.

**Table 2:** Performance values of the best ANN model.

	Training	Validation	Test	All
MSE	0.0002	0.0001	0.0018	0.0003
MAPE	0.1255	0.1345	0.5178	0.1701
R	0.9999	0.9998	0.9999	0.9999

**Fig. 3:**
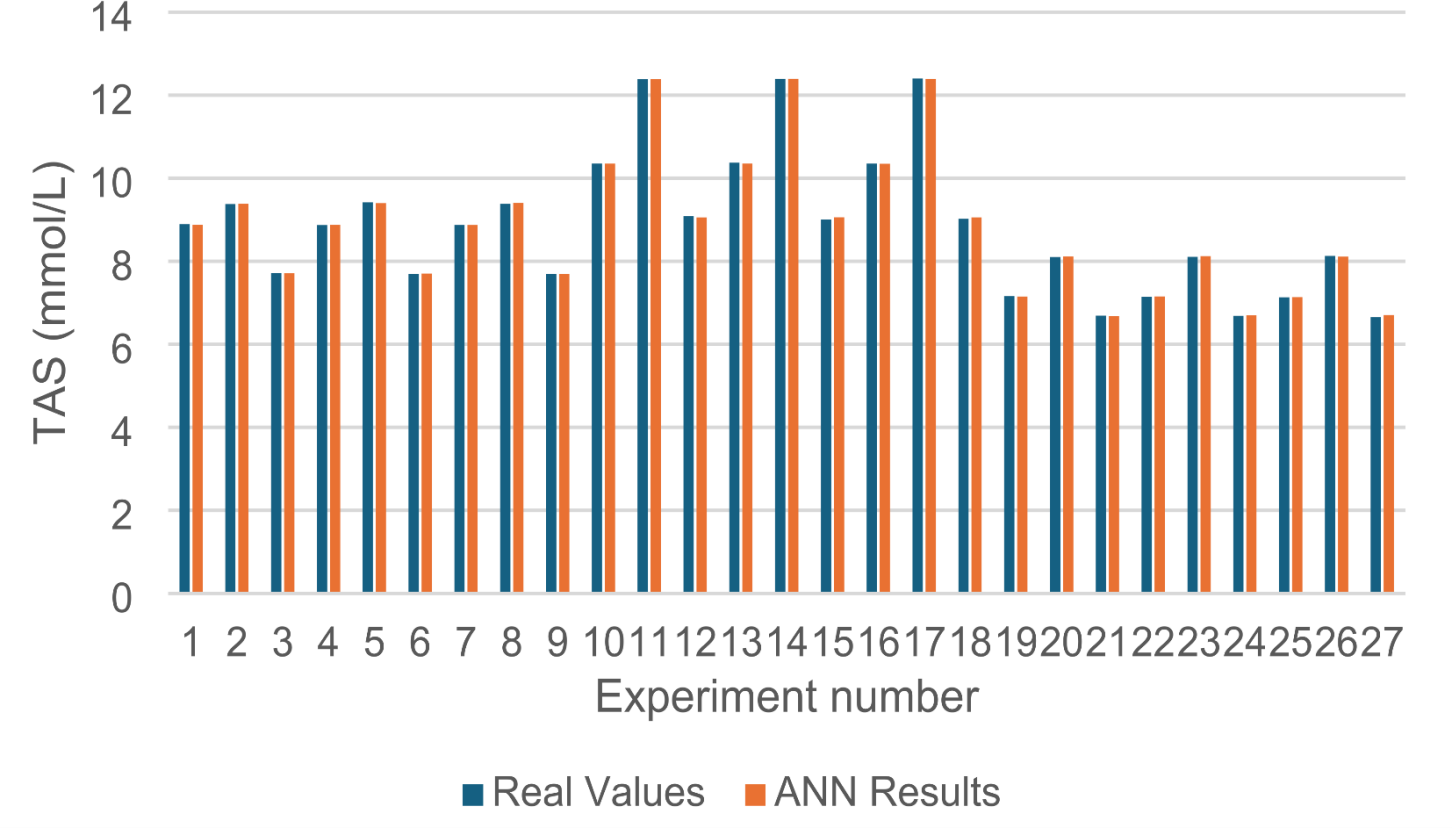
Real and predicted values of the ANN model.

The MSE, MAPE and R values of this model were calculated as 0.0003, 0.170% and 0.999 for all values, respectively. The predicted values of the model are very close to the actual values. The predicted and actual values are given in Fig. 3.

**Fig. 4:**
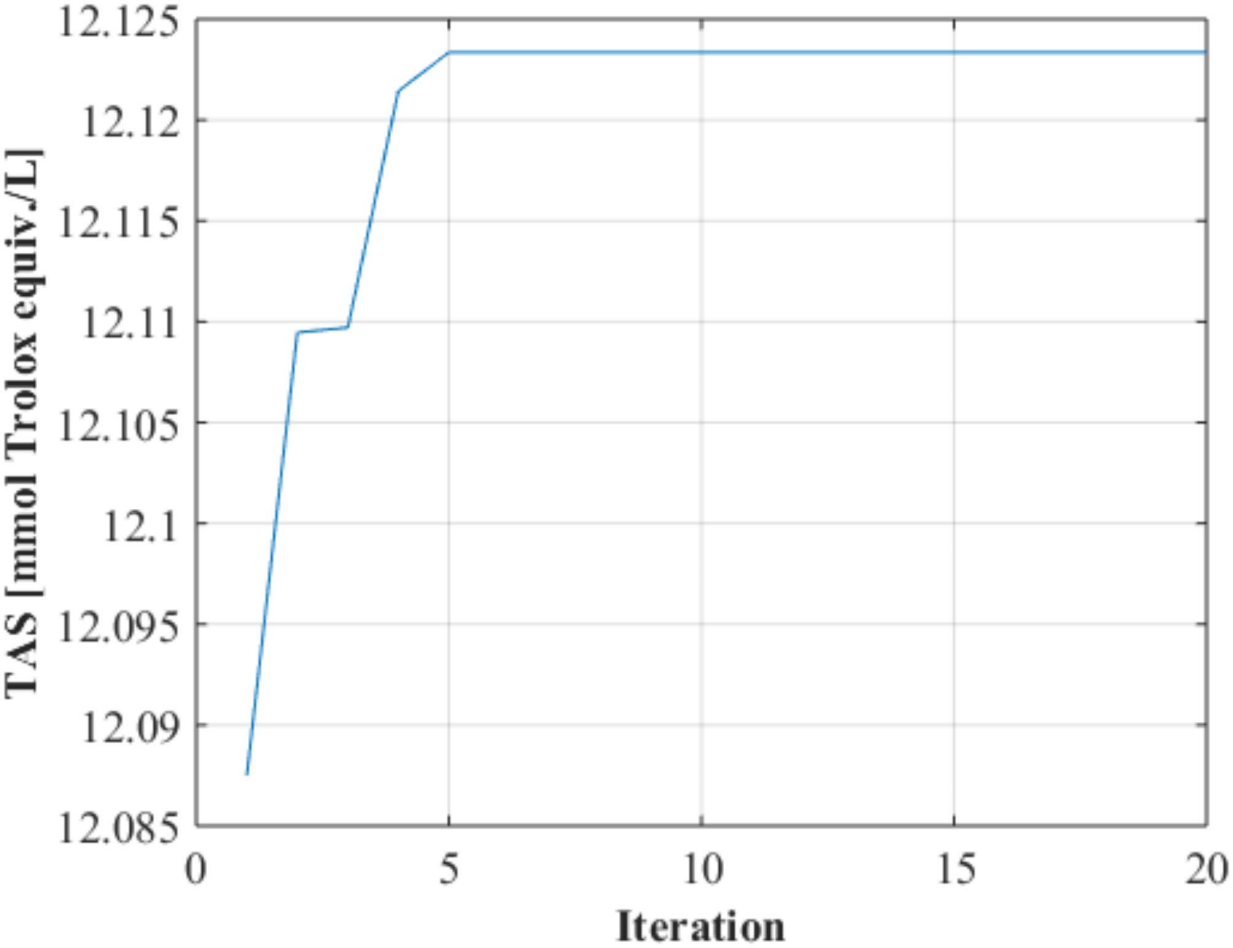
Convergence graph.

The optimization process was performed with the GA using the best ANN model selected. Among the different population numbers tested, the most suitable population number was determined as 10. After 20 iterations, the convergence graph was plotted (Fig. 4) and it was observed that the objective function value remained constant after the 5th iteration.

According to ANN-GA integration, 57.123 ˚C temperature, 12.681 h duration and 58.680 ethanol/water ratio were predicted.

### Antioxidant activity

The accumulation of a high level of free radicals, especially reactive oxygen species, in cells is defined as oxidative stress. This can disrupt cellular structures and interfere with their functioning, causing negative consequences such as DNA damage, oxidation of proteins and peroxidation of lipids^18^. Oxidative stress is a factor in the occurrence of many diseases. These include cancer, cardiovascular disease, diabetes, and neurodegenerative diseases (such as Alzheimer’s and Parkinson’s)^19^. Although free radicals are produced naturally during normal metabolism, environmental factors (e.g., smoking, air pollution, and alcohol consumption) can worsen this process. Antioxidants are substances that prevent oxidative damage by neutralizing free radicals. The body naturally produces some enzymatic antioxidants (for example, superoxide dismutase and glutathione peroxidase)^20^. In addition, dietary vitamin C, vitamin E, flavonoids and phenolic compounds also support this function^21^. In this study, the antioxidant potential of optimized extracts of the kernel part of walnut was determined. The findings are presented in Table 3.

**Table 3:** Antioxidant parameters of optimized extracts of the kernel part of walnut.

Extract	DPPH (mg Trolox Equi/g)	FRAP (mg Trolox Equi/g)	OSI	TOS (µmol/L)	TAS (mmol/L)
OE-RSM	268.81 ± 1.28^a^	313.48 ± 1.46^a^	0.034 ± 0.001^a^	3.625 ± 0.033^a^	10.764 ± 0.038^a^
OE-ANN-GA	292.31 ± 1.55^b^	346.72 ± 0.81^b^	0.045 ± 0.001^b^	5.595 ± 0.051^b^	12.578 ± 0.016^b^

^a^Means having the different superscript letter(s) in the same column are significantly different (*p* < 0.05) according to t- test, OE-RSM: Optimized extract for RSM analysis; OE-ANN-GA: Optimized extract for Artificial Neural Network-Genetic Algorithm analysis.

There are many antioxidant activity studies on the kernel part of walnut in the literature. In the reported studies, antioxidant activities of the kernel part of walnut were reported by antioxidant analysis methods such as DPPH, FRAP, ABTS and Reducing Power^22–25^. In this study, optimized extracts of the kernel part of walnut were studied by DPPH and FRAP methods, and antioxidant activities were observed. In addition, TAS, TOS, and OSI values were determined for the first time. TAS values are an indicator of the totality of antioxidant-effective compounds produced in the plant. The TOS value is an indicator of all oxidant compounds produced in the plant. The OSI value shows the percentage of oxidant compounds suppressed by antioxidant compounds^26^. In this study, optimized extracts of walnut kernel were used, and OE-ANN-GA was found to have higher antioxidant activity than OE-RSM. In contrast, the OE-ANN-GA produced more oxidant compounds than the OE-RSM. The main reason for this is thought to be that the process of obtaining the extract is longer in the OE-ANN-GA. TAS, TOS and OSI values of different plant species have been reported in the literature. The TAS value of *Tagetes patula* was reported as 5.386 mmol/L, TOS value as 8.287 µmol/L and OSI value as 0.154^27^. The TAS value of *Hypericum spectabile* was reported as 9.306 mmol/L, TOS value as 13.065 µmol/L and OSI value as 0.140^28^. The TAS value of *Sinapis arvensis* was reported as 5.232 mmol/L, TOS value as 7.564 µmol/L and OSI value as 0.146^29^. TAS value of *Alcea kurdica* was reported as 3.298 mmol/L, TOS value as 8.312 µmol/L and OSI value as 0.252^30^. In this study, both extracts of walnut kernel had higher TAS values than *T. patula*, *H. spectabile*, *S. arvensis* and *A. kurdica*. In this context, it was determined that walnut kernel has strong antioxidant activity. In addition, both extracts of walnut kernel had lower TOS and OSI values than *T. patula*, *H. spectabile*, *S. arvensis* and *A. kurdica*. These findings indicate that walnut kernel has high antioxidant capacity and has a significant potential to suppress oxidant compounds. Therefore, it is concluded that walnut kernel can be considered as a natural source of antioxidants that may provide protective effects on health.

### Enzyme Inhibition activity

Alzheimer’s disease is a neurodegenerative disorder characterized by neuronal degeneration in the brain and loss of cognitive functions. A decrease in the neurotransmitter acetylcholine plays an important role in the pathogenesis of the disease. Cholinesterase enzymes (acetylcholinesterase and butyrylcholinesterase) break down acetylcholine and terminate neurotransmission. Cholinesterase inhibitors used in Alzheimer’s treatment increase acetylcholine levels by suppressing the activity of these enzymes and supporting the preservation of cognitive functions. Therefore, cholinesterase inhibitors have an important place in the symptomatic treatment of Alzheimer’s disease^31,32^. In this study, the potential of the optimized extracts of the kernel part of walnut for the inhibition of cholinesterase enzymes was determined. The IC50 values of the findings obtained are shown in Table 4.

**Table 4:** Anticholinesterase activity of optimized extracts of the kernel part of walnut.

Sample	AChE µg/mL	BChE µg/mL
OE-RSM	12.017 ± 0.510^c^	22.283 ± 0.394^c^
OE-ANN-GA	9.393 ± 0.162^b^	19.497 ± 0.475^b^
Galantamine	5.807 ± 0.110^a^	16.643 ± 0.223^a^

^a^Means having the different superscript letter(s) in the same column are significantly different (*p* < 0.05) according to Duncan’s multiple range test, OE-RSM: Optimized extract for RSM analysis; OE-ANN-GA: Optimized extract for Artificial Neural Network-Genetic Algorithm analysis.

As a result of the analysis, it was determined that OE-ANN-GA, one of the optimized extracts of walnut kernel, exhibited stronger anticholinesterase activity compared to OE-RSM. It was also observed that they exhibited lower activities compared to galantamine, the standard used in the study. On the contrary, it is seen that they exhibit activities close to the standard. It has been reported in the literature that *J. regia* leaves exhibit high acetylcholinesterase and butyrylcholinesterase activities^33^ In another study, it was reported that the fruit parts of *J. regia* exhibited 14.43% butyrylcholinesterase activity^34^. In another study, acetylcholinesterase activity of the seed coat of *J. regia* was reported as 5.751 µg/mL and butyrylcholinesterase activity as 30.262 µg/mL^35^. Compared to these studies, the optimized extracts of the kernel part of *J. regia* used in this study showed higher effects. Identifying the enzymes that play a role in the mechanisms of disease development and inhibiting these enzymes is a fundamental step in the development of new treatment methods. Biotechnology and pharmaceutical research contribute to more effective management of diseases with approaches based on enzyme inhibition^36^. The anticholinesterase activity exhibited by Juglans regia kernel extracts in our study is thought to be related to the high phenolic compound profile it contains. The capacity of phenolic compounds such as quercetin, kaempferol, and protocatechuic acid to inhibit acetylcholinesterase and butyrylcholinesterase enzymes has been widely reported in the literature. It is suggested that these compounds may exhibit neuroprotective activities due to their antioxidant properties and hydroxyl groups that are effective in enzyme inhibition^37^. The high activity of kernel extracts may be attributed to the concentration of phenolic compounds and the bioavailability-enhancing effect of the extraction method. While these results support the therapeutic potential of *J. regia* kernel extracts on neurodegenerative diseases, they also emphasize the importance of studying the more comprehensive biological activities of this natural product.

### Antiproliferative activity

**Fig. 5:**
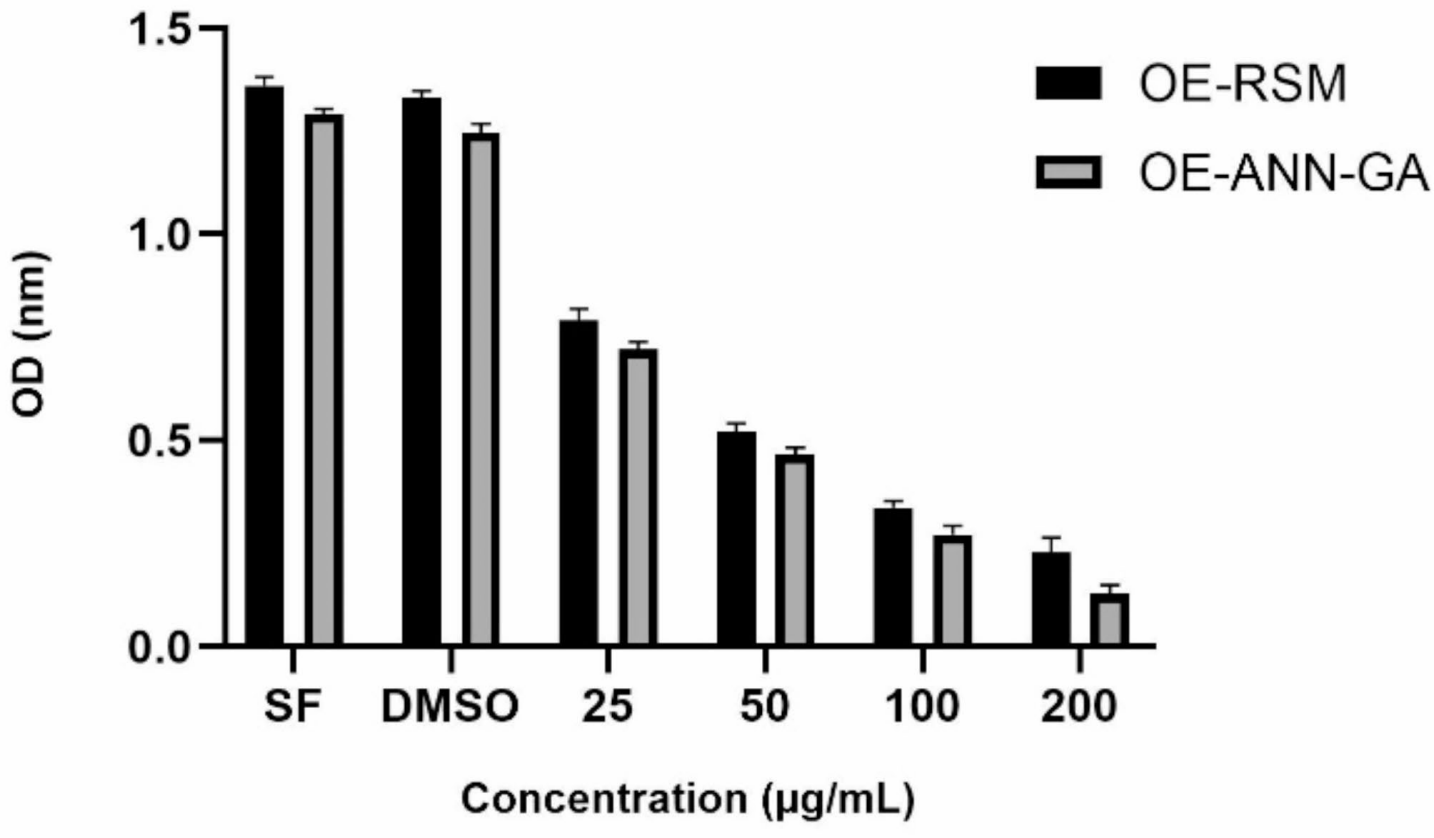
Antiproliferative activity of optimized extracts of the kernel part of walnut. (**SF Group**: Group kept only in the medium without applying any chemical substance. **DMSO Group**: Group to which only DMSO was applied together with the medium. **Extract Application Groups**: Groups to which the extract was applied at concentrations of 25, 50, 100 and 200 µg/mL), OE-RSM: Optimized extract for RSM analysis; OE-ANN-GA: Optimized extract for Artificial Neural Network-Genetic Algorithm analysis.

Plants, which are rich in bioactive components, offer significant potential in the treatment of diseases such as cancer, and especially with their antiproliferative activities, they can limit tumor growth by inhibiting cell division^38^. Many plants show selective toxicity against cancer cells and have the ability to stop the growth of cancerous cells without damaging healthy cells^39^. In our study, the activity of the optimized extract obtained from the kernel part of *J. regia* against A549 lung cancer cell line was determined. The findings obtained are shown in Fig. 5.

In the study, the activities of optimized extracts of the kernel part of *J. regia* against the A549 lung cancer cell line were determined. As a result of the analysis, it was observed that OE-ANN-GA exhibited stronger activity compared to OE-RSM. In addition, it was determined that both extracts exhibited highly potent cytotoxic activities against the A549 lung cancer cell line with increasing concentration. In the literature, it has been reported that seed, green husk, and leaf parts of Juglans regia exhibit strong antiproliferative activities against the colon cancer cell line Caco-2 and human renal cancer cell lines A-498 and 769-P^40^. In another study, it was reported that juglone obtained from *J. regia* was effective against breast (T47D), prostate colon (Colo-205 and HCT-116), skin (A-431), lung (NCI-H322 and A549) and prostate (PC-3 and DU-145) cell lines^41^. In this study, it was determined that the extracts obtained from the kernel part of *J. regia* under optimum conditions that maximized the biological activity of the extracts exhibited strong cytotoxic activities against A549 lung cancer cell line. The potent cytotoxic effect of Juglans regia kernel extracts against A549 lung cancer cell line is considered to be related to the rich profile of phenolic compounds it contains. Especially phenolic compounds such as quercetin, kaempferol, and protocatechuic acid have been widely reported in the literature to have cell proliferation-suppressing and apoptosis-inducing properties^42,43^. In the study, the fact that the extracts exhibited stronger cytotoxic effects with increasing concentrations supports the dose-dependent effects of these compounds. In addition, optimization processes may have enhanced the bioavailability of phenolic compounds, and the contribution of this on cytotoxic activity is thought to be significant. These findings suggest that *J. regia* kernel fraction can be considered not only as a nutritional source but also as an alternative natural therapeutic agent for cancer treatment.

### Phenolic contents

Plants not only offer potential treatment options to the health and pharmaceutical industries with the bioactive compounds they produce to protect themselves against environmental stress factors, but also enable useful applications in different areas by exhibiting various biological activities^44^. It can be said that these bioactive compounds have evolved as adaptation and survival strategies of plants to their natural environment, and these features also have the potential to benefit human health^45^. In the study, the phenolic contents of the optimized extracts of the kernel part of walnut were scanned on the LC-MS/MS device. The findings are shown in Table 5.

**Table 5:** Phenolic contents of optimized extracts of the kernel part of walnut.

Phenolic compounds	Ret. Time	OE-RSM (mg/kg)	OE-ANN-GA (mg/kg)
Gallic acid	1.483	5429.16 ± 2.48	6120.51 ± 2.12
Protocatechuic acid	2.060	1433.68 ± 0.88	1200.85 ± 1.56
Catechinhyrate	2.482	3596.89 ± 2.74	3625.17 ± 0.47
4-hydroxybenzoic acid	2.550	921.46 ± 0.75	1019.63 ± 0.40
Caffeic acid	2.850	9224.65 ± 0.29	13582.76 ± 1.64
Vanillic acid	2.853	6823.12 ± 2.19	7256.95 ± 0.60
Syringic acid	2.973	4220.36 ± 0.87	4263.29 ± 1.89
2-hydoxycinamic acid	3.553	587.58 ± 0.84	1274.60 ± 0.66
Resveratrol	3.632	136.42 ± 0.97	782.66 ± 1.48
2-hyroxy1,4 naphthaquinone	3.654	2194.16 ± 3.40	633.89 ± 0.72
Myricetin	3.725	3455.89 ± 0.71	3901.15 ± 3.00
Quercetin	4.000	20195.47 ± 1.63	30154.88 ± 1.37
Kaempferol	4.222	25136.42 ± 0.47	29420.69 ± 1.07

*OE-RSM: Optimized extract for RSM analysis; OE-ANN-GA: Optimized extract for Artificial Neural Network-Genetic Algorithm analysis.

In the literature, the presence of many phenolic compounds such as pyrogallol, p-hydroxybenzoic acid, vanillic acid, ethyl gallate, protocatechuic acid, gallic acid and 3,4,8,9,10-pentahydroxydibenzo[b, d]pyran-6-one, ferulic acid, catechin, p-coumaric acid, Ellagic acid, pyrocathechin, epicathechin, rutin, syringic acid, juglone, cinnamic acid have been reported in the kernel part of *J. regia*^23,46,47^. In our study, 13 phenolic compounds were determined in the optimum extracts obtained from the kernel part of *J. regia*. The highest amount of phenolic compound detected in OE-ANN-GA was quercetin. In OE-RSM extract, it was kaempferol. In addition, protocatechuic acid and 2-hyroxy1,4 naphthaquinone compounds were determined higher in OE-RSM compared to OE-ANN-GA. On the other hand, gallic acid, catechinhyrate, 4-hydroxybenzoic acid, caffeic acid, vanillic acid, syringic acid, 2-hydoxycinamic acid, resveratrol, myricetin, quercetin and kaempferol compounds were determined higher in OE-ANN-GA. The different distribution of phenolic compounds in the kernel extracts of Juglans regia may be due to the parameters of the applied extraction method and the chemical properties of the compounds. In our study, the differences in the compounds observed in the extracts can be explained by the effects of temperature, solvent percentages and extraction times on the release of compounds. For example, the higher levels of flavonoids such as quercetin and kaempferol in OE-ANN-GA indicate that the solubility properties of these compounds are more compatible with the second extraction parameters. Better solubility with the properties of the solvent used in the extraction of natural products may contribute to these results. These differences emphasize the effect of the extraction method on the phenolic compound profile and the importance of correct optimization.

## Conclusion

In this study, it was determined that the extracts obtained from the kernel part of *J. regia* under optimum conditions showed high biological activity. As a result of the experiments, it was determined that the antioxidant capacity of the extracts was high, their antiproliferative effects against the A549 lung cancer cell line were strong, and they showed acetylcholinesterase and butyrylcholinesterase inhibitory activities. In addition, it was determined that important phenolic compounds such as quercetin and kaempferol were found at high levels in the extracts. These findings also reveal the contribution of the phenolic compound-rich structure of the Juglans regia kernel extracts to biological activities. It is promising in terms of developing natural and effective treatment alternatives, especially against important health problems such as neurodegenerative diseases and cancer. In further studies, more detailed examination of the molecular mechanisms and effective doses of these extracts may pave the way for new approaches towards clinical applications.

## Data Availability

The datasets used and/or analysed during the current study available from the corresponding author on reasonable request.

## References

[1] Mohammed, F. S., Sevindik, M., Uysal, İ., Çesko, C. & Koraqi, H. Chemical composition, biological activities, uses, nutritional and mineral contents of Cumin (Cuminum cyminum). *Measurement: Food*, 100157 (2024).

[2] Cedillo-Cortezano, M. et al. Use of medicinal plants in the process of wound healing: a literature review. *Pharmaceuticals* **17**, 303 (2024). 38543089 10.3390/ph17030303 PMC10975678

[3] Guo, M. et al. Strategies on biosynthesis and production of bioactive compounds in medicinal plants. *Chin. Herb. Med.* **16**, 13–26 (2024). 38375043 10.1016/j.chmed.2023.01.007 PMC10874775

[4] Delaviz, H., Mohammadi, J., Ghalamfarsa, G. & Mohammadi, B. Farhadi, N. A review study on phytochemistry and Pharmacology applications of Juglans regia plant. *Pharmacogn. Rev.* **11**, 145 (2017). 28989250 10.4103/phrev.phrev_10_17 PMC5628521

[5] Quratul-ain, M. S. & Siddiqui, M. Medicinal and nutritional importance of Juglans regia Linn. On human health. *Med. Plants their Bioactive Compd. Hum. Health* **1**, 165 (2024).

[6] Bhardwaj, A., Singh, A., Patnaik, R. S., Bhardwaj, S. & Juglans Regia, L. A review of its traditional uses phytochemistry and therapeutic applications. *J. Pharm. Negat. Results* **14**, 11–16 (2023).

[7] Li, B. et al. Traditional applications, ethnopharmacology, and phytochemistry of walnut green husk (Juglans regia L.): A review. *Nat. Prod. Commun.* **19**, 1934578X241262156 (2024).

[8] Hayes, D., Angove, M. J., Tucci, J. & Dennis, C. Walnuts (Juglans regia) chemical composition and research in human health. *Crit. Rev. Food Sci. Nutr.* **56**, 1231–1241 (2016). 25747270 10.1080/10408398.2012.760516

[9] Jaiswal, B. S. & Tailang, M. Juglans Regia: A review of its traditional uses phytochemistry and Pharmacology. *Indo Am. J. Pharm. Res.* **7**, 390–398 (2017).

[10] Bourais, I. et al. A review on medicinal uses, nutritional value, and antimicrobial, antioxidant, anti-inflammatory, antidiabetic, and anticancer potential related to bioactive compounds of J. regia. *Food Reviews Int.* **39**, 6199–6249 (2023).

[11] Ong, M. Y., Nomanbhay, S., Kusumo, F., Raja Shahruzzaman, R. M. H. & Shamsuddin, A. H. Modeling and optimization of microwave-based bio-jet fuel from coconut oil: investigation of response surface methodology (RSM) and artificial neural network methodology (ANN). *Energies* **14**, 295 (2021).

[12] Erel, O. A novel automated direct measurement method for total antioxidant capacity using a new generation, more stable ABTS radical cation. *Clin. Biochem.* **37**, 277–285 (2004). 15003729 10.1016/j.clinbiochem.2003.11.015

[13] Erel, O. A new automated colorimetric method for measuring total oxidant status. *Clin. Biochem.* **38**, 1103–1111 (2005). 16214125 10.1016/j.clinbiochem.2005.08.008

[14] Sevindik, M. The novel biological tests on various extracts of cerioporus varius. *Fresenius Environ. Bull.* **28**, 3713–3717 (2019).

[15] Sevindik, M., Gürgen, A., Khassanov, V. T. & Bal, C. Biological activities of ethanol extracts of hericium erinaceus obtained as a result of optimization analysis. *Foods* **13**, 1560 (2024). 38790860 10.3390/foods13101560 PMC11121622

[16] Ellman, G. L., Courtney, K. D., Andres Jr, V. & Featherstone, R. M. A new and rapid colorimetric determination of acetylcholinesterase activity. *Biochem. Pharmacol.* **7**, 88–95 (1961). 13726518 10.1016/0006-2952(61)90145-9

[17] Bal, C., Akgul, H., Sevindik, M., Akata, I. & Yumrutas, O. Determination of the anti-oxidative activities of six mushrooms. *Fresenius Envir Bull.* **26**, 6246–6252 (2017).

[18] Sevindik, M., Mohammed, F. S. & Uysal, I. Autism: plants with neuro-psychopharmacotherapeutic potential. *Prospects Pharm. Sci.* **21**, 38–48 (2023).

[19] Jomova, K. et al. Reactive oxygen species, toxicity, oxidative stress, and antioxidants: chronic diseases and aging. *Arch. Toxicol.* **97**, 2499–2574 (2023). 37597078 10.1007/s00204-023-03562-9 PMC10475008

[20] Uysal, I. Total phenolic and flavonoid contents and antioxidant, antimicrobial and antiproliferative activities of Polycarpon tetraphyllum. *Kuwait J. Sci.* **50**, 322–325 (2023).

[21] Olufunmilayo, E. O., Gerke-Duncan, M. B. & Holsinger, R. D. Oxidative stress and antioxidants in neurodegenerative disorders. *Antioxidants* **12**, 517 (2023). 36830075 10.3390/antiox12020517 PMC9952099

[22] Samaranayaka, A. G., John, J. A. & Shahidi, F. Antioxidant activity of english walnut (Juglans regia L). *J. Food Lipids* **15**, 384–397 (2008).

[23] Zhang, Z., Liao, L., Moore, J., Wu, T. & Wang, Z. Antioxidant phenolic compounds from walnut kernels (Juglans regia L). *Food Chem.* **113**, 160–165 (2009).

[24] Amin, F., Masoodi, F., Baba, W. N., Khan, A. A. & Ganie, B. A. Effect of different ripening stages on walnut kernel quality: antioxidant activities, lipid characterization and antibacterial properties. *J. Food Sci. Technol.* **54**, 3791–3801 (2017). 29085121 10.1007/s13197-017-2776-4 PMC5643792

[25] Wu, S. et al. Phenolic profiles and antioxidant activities of free, esterified and bound phenolic compounds in walnut kernel. *Food Chem.* **350**, 129217 (2021). 33607410 10.1016/j.foodchem.2021.129217

[26] Sabik, A. E., Sevindik, M., Mohammed, F. S. & Dogan, M. A. New natural source against A549 lung Cancer cells Anthemis cotula and its biological activities and phenolic contents. *Pharm. Chem. J.* **58**, 1134–1140 (2024).

[27] Seğmenoğlu, M. S. & Sevindik, M. Antioxidant and antimicrobial potentials of functional food Arum Dioscoridis. *Sigma J. Eng. Nat. Sci.* **42**, 116–120 (2024).

[28] Gürgen, A., Sevindik, M., Krupodorova, T., Uysal, I. & Unal, O. Biological activities of Hypericum spectabile extract optimized using artificial neural network combined with genetic algorithm application. *BMC Biotechnol.* **24**, 83 (2024). 39468527 10.1186/s12896-024-00914-w PMC11520853

[29] Mohammed, F., Sevindik, M. & Uysal, I. Total phenolic, flavonoid, protein contents and biological activities of wild mustard. *Acta Aliment.* **52**, 449–457 (2023).

[30] Mohammed, F., Sevindik, M., Uysal, I., Sevindik, E. & Akgül, H. A natural material for suppressing the effects of oxidative stress: biological activities of Alcea Kurdica. *Biology Bull.* **49**, S59–S66 (2022).

[31] Breijyeh, Z. & Karaman, R. Comprehensive review on Alzheimer’s disease: causes and treatment. *Molecules* **25**, 5789 (2020). 33302541 10.3390/molecules25245789 PMC7764106

[32] Haake, A., Nguyen, K., Friedman, L., Chakkamparambil, B. & Grossberg, G. T. An update on the utility and safety of cholinesterase inhibitors for the treatment of Alzheimer’s disease. *Exp. Opin. Drug Saf.* **19**, 147–157 (2020). 10.1080/14740338.2020.1721456 31976781

[33] Karakaya, S., Koca, M., Yeşilyurt, F. & Hacımüftüoğlu, A. Antioxidant and anticholinesterase activities of Juglans regia L. growing in Turkey. *J. Fac. Pharm. Ankara Univ.* **43**, 230–238 (2019).

[34] Orhan, I. E., Suntar, I. P. & Akkol, E. K. In vitro neuroprotective effects of the leaf and fruit extracts of Juglans regia L.(walnut) through enzymes linked to Alzheimer’s disease and antioxidant activity. *Int. J. Food Sci. Nutr.* **62**, 781–786 (2011). 21627404 10.3109/09637486.2011.585964

[35] Palabıyık, E., Uğuz, H., Aşkın, H., Aşkın, S. & Akıncıoğlu, H. Walnut seed coat (Juglans regia L.), a plant effective in human health: antioxidant activity and in rats nephroprotective effect. *Res. Agricultural Sci.* **55**, 89–104 (2024).

[36] Świątek, Ł. et al. LC-ESI-QTOF-MS/MS analysis, cytotoxic, antiviral, antioxidant, and enzyme inhibitory properties of four extracts of Geranium pyrenaicum burm. F.: A good gift from the natural treasure. *Int. J. Mol. Sci.* **22**, 1–26 (2021). 10.3390/ijms22147621 PMC8307321 34299238

[37] Szwajgier, D., Baranowska-Wojcik, E. & Borowiec, K. Phenolic acids exert anticholinesterase and cognition-improving effects. *Curr. Alzheimer Res.* **15**, 531–543 (2018). 29189157 10.2174/1567205014666171128102557

[38] Khan, M. I. et al. Anticancer properties of medicinal plants and their bioactive compounds against breast cancer: a review on recent investigations. *Environ. Sci. Pollut. Res.* **29**, 24411–24444 (2022). 10.1007/s11356-021-17795-7 35064485

[39] Siddiqui, A. J. et al. Plants in anticancer drug discovery: from molecular mechanism to chemoprevention. *BioMed Research International* 2022, 5425485 (2022). 10.1155/2022/5425485 PMC8906971 35281598

[40] Carvalho, M. et al. Human cancer cell antiproliferative and antioxidant activities of Juglans regia L. *Food Chem. Toxicol.* **48**, 441–447 (2010). 19883717 10.1016/j.fct.2009.10.043

[41] Zhang, X. B., Zou, C. L., Duan, Y. X., Wu, F. & Li, G. Activity guided isolation and modification of juglone from Juglans regia as potent cytotoxic agent against lung cancer cell lines. *BMC Complement. Altern. Med.* **15**, 1–8 (2015). 26530090 10.1186/s12906-015-0920-0 PMC4632350

[42] Dai, J. & Mumper, R. J. Plant phenolics: extraction, analysis and their antioxidant and anticancer properties. *Molecules* **15**, 7313–7352 (2010). 20966876 10.3390/molecules15107313 PMC6259146

[43] El-Ansari, M. A., Ibrahim, L. F. & Sharaf, M. Natural phenolics: a source of anticancer agents. *Egypt. Pharm. J.* **18**, 1–7 (2019).

[44] Zhang, H., Jiang, F., Li, L., Liu, X. & Yan, J. K. Recent advances in the bioactive polysaccharides And other key components from Phellinus spp. And their Pharmacological effects: A review. *Int. J. Biol. Macromol.* **222**, 3108–3128 (2022). 36243155 10.1016/j.ijbiomac.2022.10.085

[45] El-Chaghaby, G. A. et al. Genus hypericum: general properties, chemical contents and biological activities. *Egypt. J. Bot.* **64**, 1–26 (2024).

[46] Bujdoso, G., Vegvari, G., Hajnal, V., Ficzek, G. & Magdolna, T. Phenolic profile of the kernel of selected Persian walnut (Juglans regia L.) cultivars. *Notulae Botanicae Horti Agrobotanici Cluj-Napoca*. **42**, 24–29 (2014).

[47] Slatnar, A., Mikulic-Petkovsek, M., Stampar, F., Veberic, R. & Solar, A. Identification and quantification of phenolic compounds in kernels, oil and Bagasse pellets of common walnut (Juglans regia L). *Food Res. Int.* **67**, 255–263 (2015). 10.1016/j.foodres.2014.08.009 30011716

